# Bacterial Secondary Metabolite Biosynthetic Potential in Soil Varies with Phylum, Depth, and Vegetation Type

**DOI:** 10.1128/mBio.00416-20

**Published:** 2020-06-16

**Authors:** Allison M. Sharrar, Alexander Crits-Christoph, Raphaël Méheust, Spencer Diamond, Evan P. Starr, Jillian F. Banfield

**Affiliations:** a Department of Earth and Planetary Science, University of California, Berkeley, Berkeley, California, USA; b Department of Plant and Microbial Biology, University of California, Berkeley, Berkeley, California, USA; c Innovative Genomics Institute, Berkeley, California, USA; University of British Columbia

**Keywords:** metagenomics, secondary metabolism, soil microbiology

## Abstract

Bacteria isolated from soils are major sources of specialized metabolites, including antibiotics and other compounds with clinical value that likely shape interactions among microbial community members and impact biogeochemical cycles. Yet, isolated lineages represent a small fraction of all soil bacterial diversity. It remains unclear how the production of specialized metabolites varies across the phylogenetic diversity of bacterial species in soils and whether the genetic potential for production of these metabolites differs with soil depth and vegetation type within a geographic region. We sampled soils and saprolite from three sites in a northern California Critical Zone Observatory with various vegetation and bedrock characteristics and reconstructed 1,334 metagenome-assembled genomes containing diverse biosynthetic gene clusters (BGCs) for secondary metabolite production. We obtained genomes for prolific producers of secondary metabolites, including novel groups within the *Actinobacteria*, *Chloroflexi*, and candidate phylum “*Candidatus* Dormibacteraeota.” Surprisingly, one genome of a candidate phyla radiation (CPR) bacterium coded for a ribosomally synthesized linear azole/azoline-containing peptide, a capacity we found in other publicly available CPR bacterial genomes. Overall, bacteria with higher biosynthetic potential were enriched in shallow soils and grassland soils, with patterns of abundance of BGC type varying by taxonomy.

## Abstract (precis)

Microbes produce specialized compounds to compete or communicate with one another and their environment. Some of these compounds, such as antibiotics, are also useful in medicine and biotechnology. Historically, most antibiotics have come from soil bacteria which can be isolated and grown in the lab. Though the vast majority of soil bacteria cannot be isolated, we can extract their genetic information and search it for genes which produce these specialized compounds. These understudied soil bacteria offer a wealth of potential for the discovery of new and important microbial products. Here, we identified the ability to produce these specialized compounds in diverse and novel bacteria in a range of soil environments. This information will be useful to other researchers who wish to isolate certain products. Beyond their use to humans, understanding the distribution and function of microbial products is key to understanding microbial communities and their effects on biogeochemical cycles.

## INTRODUCTION

Many soil microbes synthesize secondary metabolite molecules that play important ecological roles in their complex and heterogeneous microenvironments. Secondary (or “specialized”) metabolites are auxiliary compounds that microbes produce which are not required for normal cell growth but which benefit the cells in other ways. These compounds can have roles in nutrient acquisition, communication, and inhibition or in other interactions with surrounding organisms or the environment (1). Examples of these molecules include antibiotics (1), siderophores (2), quorum-sensing molecules (3), immunosuppressants (4), and degradative enzymes (5).

Secondary metabolites are of interest for both their ecological and biogeochemical effects, as well as their potential for use in medicine and biotechnology. Antibiotics are a class of secondary metabolites with obvious importance to humanity. Historically, antibiotic discovery relied on being able to culture organisms from the environment; however, the vast majority of environmental taxa cannot be cultured using current methods. Most known antibiotics are from cultured members of *Actinobacteria*, *Proteobacteria*, and *Firmicutes* (6). Because soil microbial communities are so diverse and most microbial taxa in soil have not been well described (7), they offer a wealth of potential for the discovery of new and important microbial products.

Secondary metabolites are produced by biosynthetic gene clusters (BGCs), groups of colocated genes that function together to build a molecule. Nonribosomal peptide synthetases (NRPSs) and polyketide synthases (PKSs) are two of the largest classes of BGCs, encompassing most known antibiotics and antifungals (6). NRPSs are characterized by condensation (CD) and adenylation (AD) domains (8), and PKSs contain ketosynthase (KS) domains and a variety of other enzymatic domains (9). These characteristic domains can be used to identify novel NRPS and PKS gene clusters, and their abundances can be used as a proxy for biosynthetic potential (10).

Little is known about how environmental variables impact the distribution of secondary metabolites in soil. Recent studies of microbial biosynthetic potential in soil have utilized amplicon sequencing of NRPS and PKS domains (11–15). One study involving soils from a variety of environments demonstrated that NRPS and PKS domain richness was high in arid soils and low in forested soils (11). Others showed that the compositions of these domains correlated with latitude (12) and vegetation (13) at the continental scale and were distinct between urban and nonurban soils (14). Because those studies relied on degenerate PCR primers designed for known domains, only sequences similar to known domains were able to be recovered. In contrast, genome-resolved metagenomics is able to recover divergent sequences within their genomic and phylogenetic context. Recently, this approach revealed abundant biosynthetic loci in *Acidobacteria*, *Verrucomicrobia*, *Gemmatimonadetes*, and the candidate phylum “*Candidatus* Rokubacteria” (16).

Here, we hypothesized that ecological forces such as soil depth, overlying vegetation, bedrock lithology, and rainfall select for bacteria that rely to different extents on secondary metabolites involved in interorganism competition and environmental interaction. Because it is expected that changing environmental parameters would alter community composition and functions, it is important to test this hypothesis, in part to guide future targeted isolation experiments. We tested this hypothesis by sampling soils and saprolites with five different combinations of overlying vegetation and underlying bedrock lithology within three ecosystems: a meadow grassland and a nearby forested hillslope that share bedrock lithology and a hilly grassland with scattered oak trees and differing bedrock characteristics. We reconstructed genomes from the 129 resulting metagenomes and searched them for BGCs. A subset of the genomes from meadow grassland soils analyzed here were previously reported in studies that both reported on novel BGCs (16) and demonstrated that soil depth and soil moisture affect microbial community structure and function (17, 18). We present a comparative analysis of the biosynthetic potential of bacteria from many phyla and report how soil microbiology and secondary metabolic potential vary with soil type and environmental conditions. Metagenomic studies such as this one have the ability to identify new environmental and taxonomic targets key to the understanding of secondary metabolism ecology and for the development of microbial natural products of human interest.

(This article was submitted to an online preprint archive [19]).

## RESULTS

### Microbial community structure across the Eel River CZO.

**FIG 1:**
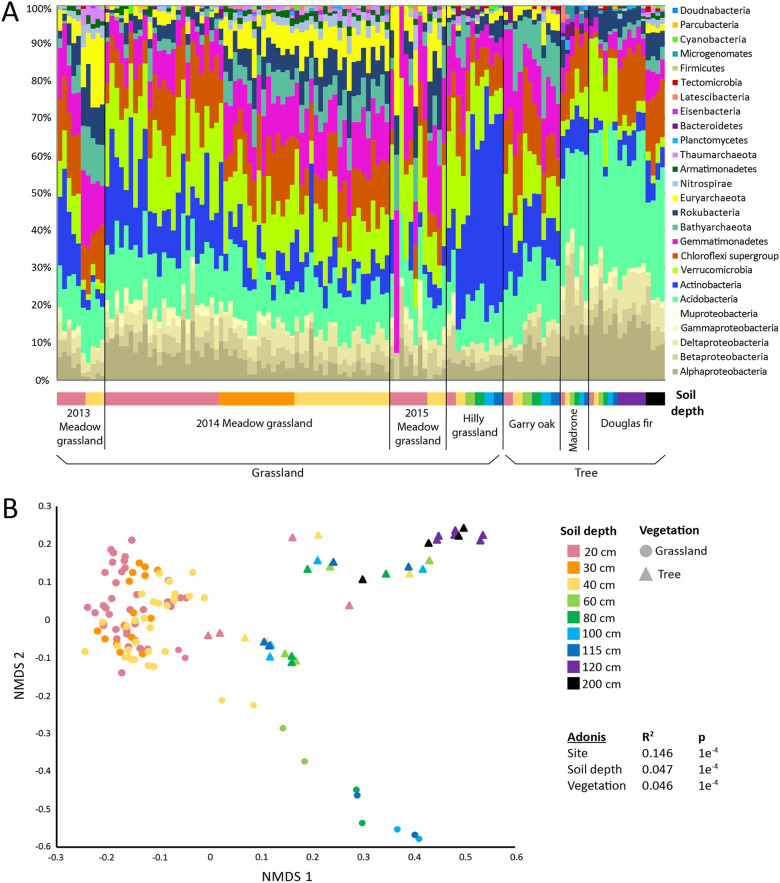
Microbial community structure across the Eel River CZO. (A) Relative abundances of microbial phyla making up >1% of the community based on coverage of ribosomal protein S3 (rpS3)-containing contigs across the Eel River CZO metagenomes (*n *= 129). Samples are grouped by site and either year sampled or environment (black vertical lines) and then by soil depth (colored bar at bottom; see legend in panel B. (B) NMDS ordination (stress = 0.0597) of microbial community composition derived from read mapping of dereplicated rpS3-containing contigs and Bray-Curtis dissimilarities. Each point represents one metagenomic sample (*n *= 129). Data representing relative variable importance (R^2^) and significance (p) calculated by PERMANOVA (Adonis) are displayed.

To compare microbial community compositions across samples with various depths, vegetations, and bedrock lithologies, assembled sequences of ribosomal protein S3 (rpS3) were used as marker genes for identifying different taxa. RpS3 is a universal single-copy gene, assembles well from metagenomic data, and is recovered more frequently than whole genomes, which allows a more inclusive view of microbial communities (20). Our rpS3 analysis indicates that microbial communities across the Eel River Critical Zone Observatory (CZO) were generally dominated by the same bacterial phyla (Proteobacteria, *Acidobacteria*, *Actinobacteria*, *Verrucomicrobia*, *Chloroflexi*, and *Gemmatimonadetes*) but that community compositions were distinct between sampling sites and at different depths within the same site (Fig. 1A). *Archaea* were abundant, making up as much as 30% of the community in some samples. Some candidate phyla radiation (CPR) bacteria were present at low abundance (usually <1% of the community) in most samples except in the meadow grassland samples. *Acidobacteria* were very abundant in Douglas fir and Madrone soil, whereas *Actinobacteria* were very abundant in the hilly grassland soil, especially at depth. At the meadow grassland, *Archaea*, “*Candidatus* Rokubacteria,” and *Nitrospirae* were more abundant with depth.

Of the environmental characteristics considered, sampling site, soil depth, and vegetation all had significant effects on microbial community composition. When controlling for the confounding variable of sampling site (marginal influence *R*^2^ = 0.146, *P* = 1e^−4^), soil depth and vegetation were found to have similar degrees of marginal influence (*R*^2^ = 0.047 and *P* = 1e^−4^ and *R*^2^ = 0.046 and *P* = 1e^−4^, respectively) (Fig. 1B). These influences are reflected in the clustering of sample points in the nonmetric multidimensional scaling (NMDS) by vegetation, with patterns of spread related to depth. The ordination showed no apparent effect of bedrock lithology or natural or artificial rainfall on sample clustering.

### Genome recovery and biosynthetic potential across taxonomic groups.

We reconstructed 15,473 genomic bins from the 129 metagenomes. This set was narrowed to 3,895 metagenome-assembled genomes (MAGs) after consideration of completeness and contamination and was dereplicated to a final set of 1,334 MAGs used in subsequent analyses (see Table S2 in the supplemental material), 944 of which were previously unpublished. Of the 1,315 non-CPR MAGs, 374 are considered high-quality drafts and 941 are considered medium-quality drafts (21) (Table S2).

**FIG 2:**
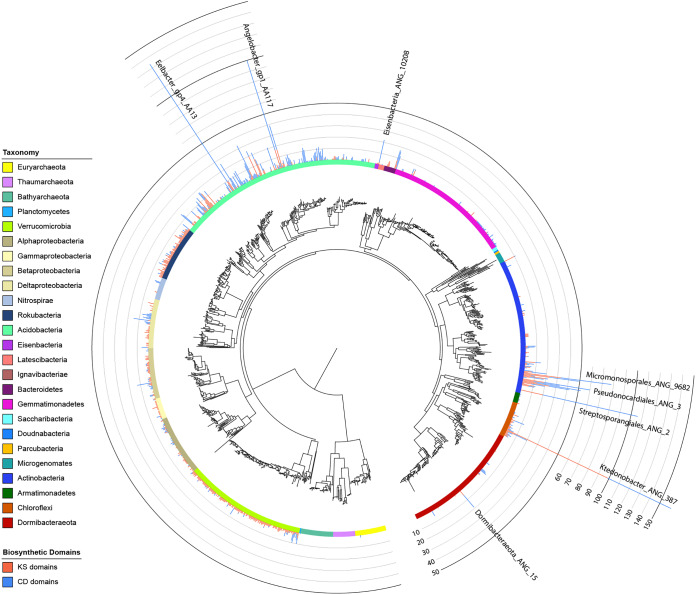
Concatenated ribosomal protein tree of dereplicated genomes. The maximum-likelihood tree is based on the concatenation of 16 ribosomal proteins from genomes from all 129 metagenomic samples that passed thresholds of >70% complete and <10% contamination, according to CheckM (*n *= 1,334). Each colored ring indicates a taxonomic group. Stacked bar plots show the amounts of KS (red) and CD (blue) domains identified by antiSMASH in each genome. Figure S1 is a higher-resolution version of this figure with genome names and bootstrap information included.

**FIG 3:**
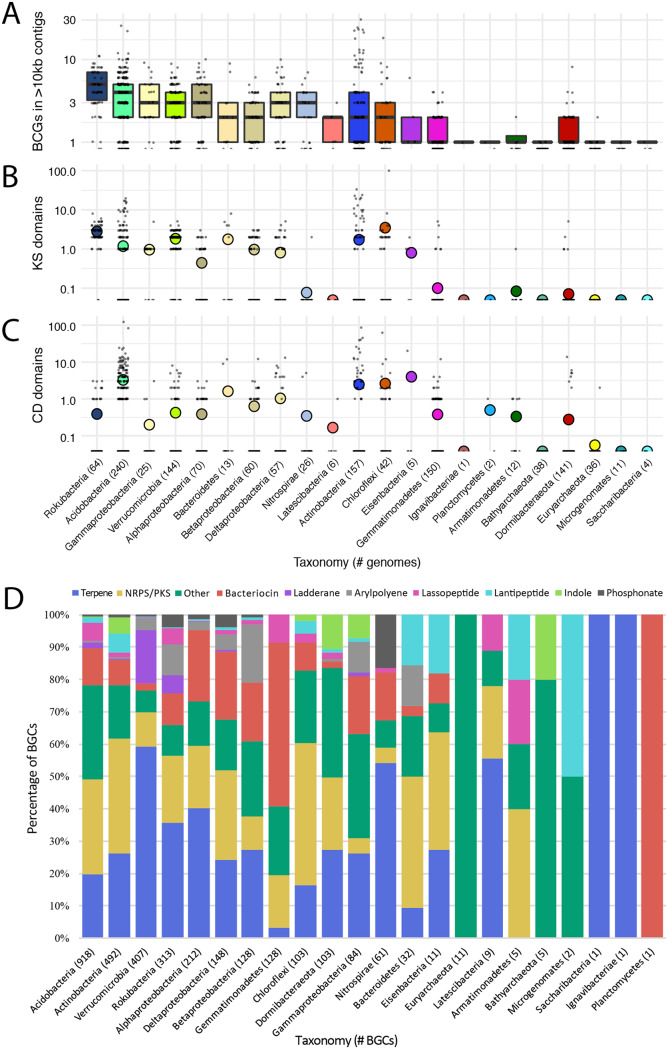
Biosynthetic gene clusters (BGCs) and key domains across taxonomic groups. Taxonomic groups with no BGCs, KS domains, or CD domains were excluded. (A) BGCs per genome on contigs of >10 kb, as identified by antiSMASH (log_10_ scale). Taxonomic groups are ordered by decreasing median value (line within box plot). The number of genomes per group is indicated in parentheses. (B and C) KS domains (B) and CD domains (C) per genome, as called by antiSMASH (log_10_ scale). Mean group value data are represented as a colored dot. (D) Percentage of BGC types within taxonomic groups, as called by antiSMASH. Known types present at <1% of all BGCs were grouped into the “Other” category. Total number of BGCs per group is indicated in parentheses.

Overall, 3,175 BGCs were identified on contigs of >10 kb within the set of 1,334 dereplicated genomes (Table S3). These genomes belonged to 22 different bacterial phylum-level groups, most of which showed some level of biosynthetic potential, and to three archaeal phyla (Fig. 2; see also Fig. S1 in the supplemental material). Bacteria from certain phyla, such as the candidate phylum “*Candidatus* Rokubacteria,” consistently had moderate numbers of BGCs in their genomes (Fig. 3A). Other phyla, such as *Actinobacteria* and *Chloroflexi*, had lower median values but contained individual genomes with exceptionally high numbers of BGCs (Fig. 3A). It was previously shown that NRPS/PKS gene clusters often result in fragmented assemblies from short reads (22). While we saw 1,100 CD and 939 KS domains in total on contigs of >10 kb across our genomes, we also saw 555 CD and 417 KS domains on contigs of <10 kb, indicating that a large fraction of BGCs in the genomes of these microbes may not have been analyzed here. Average amounts of KS and CD domains per genome (on contigs of any size) also varied by phylum (Fig. 3B and C). “*Candidatus* Rokubacteria” organisms commonly had moderate amounts of KS domains but rarely many CD domains, whereas *Acidobacteria* more often had large amounts of CD domains.

10.1128/mBio.00416-20.1 FIG S1 High-resolution version of Fig. 3 with genome names and bootstrap information (concatenated ribosomal protein tree of dereplicated genomes). The maximum-likelihood tree is based on the concatenation of 16 ribosomal proteins from genomes from all 129 metagenomic samples that passed the criteria of thresholds of >70% complete and <10% contamination, according to CheckM (*n *= 1,334). The colored ring indicates taxonomic group. Stacked bar plots show the amounts of KS (red) and CD (blue) domains identified by antiSMASH in each genome. Black dots on branches represent bootstrap values of at least 90%. Download FIG S1, PDF file, 0.4 MB. Copyright © 2020 Sharrar et al. 2020 Sharrar et al. This content is distributed under the terms of the Creative Commons Attribution 4.0 International license.

The genome with the most BGCs (23) was actinobacterium Streptosporangiales_ANG_2, found at a depth of 40 cm in hilly grassland soil (Fig. 2). A *Chloroflexi* genome collected from a depth of 20 cm in meadow grassland soil (Ktedonobacter_ANG_387) had the most KS domains (99 in total). The genome with the most CD domains (124) was a previously reported *Acidobacteria* genome from a depth of 20 cm in meadow grassland soil (Eelbacter_gp4_AA13 [16]). All genomes with unusually high numbers of BGCs (>15) or KS domains (>10) were classified as *Actinobacteria*, *Acidobacteria*, or *Chloroflexi*. *Acidobacteria*, *Actinobacteria*, *Chloroflexi*, *Gemmatimonadetes*, Deltaproteobacteria, *Betaproteobacteria*, *Bacteroidetes*, and the candidate phyla “*Candidatus* Eisenbacteria” and “*Candidatus* Dormibacteraeota” had members whose BGCs contained >10 CD domains (Table S2).

The most common types of BGCs identified in this study were terpenes, NRPS/PKS clusters, and bacteriocins. The types of BGCs present within genomes depended on the taxonomic group. While ladderanes, arylpolyenes, lassopeptides, lantipeptides, indoles, and phosphonates were less common overall, some phyla had higher proportions of certain types of BGCs (Fig. 3D). For example, bacteriocins were particularly prominent in the genomes of *Gemmatimonadetes*, ladderanes were well represented among clusters in *Verrucomicrobia*, and phosphonates were most abundant in *Nitrospirae*.

Several genomes from new clades in the *Actinobacteria* possessed a high number of large NRPS and PKS gene clusters (Fig. S2). Those genomes with recovered 16S rRNA genes indicated that they were novel at least at the species level (Table S4). Five genomes of order *Micromonosporales* were recovered from both grasslands in the study, and two genomes of order *Pseudonocardiales* were found in the Garry oak samples. The *Micromonosporales* and *Pseudonocardiales* genomes encode an array of impressively large BGCs with little similarity to known BGCs from *Actinobacteria* in the MiBIG database (Fig. S2).

10.1128/mBio.00416-20.2 FIG S2 Concatenated ribosomal protein tree of *Actinobacteria*. The maximum-likelihood tree is based on the concatenation of 16 ribosomal proteins from all *Actinobacteria* genomes from this study and one reference genome from each genus on NCBI. Colored squares show which sample set the genome is from, and stacked bar plots show the amounts of KS (red) and CD (blue) domains identified by antiSMASH in each genome. Operon diagrams are shown for select BGCs (labelled 1 to 4). Download FIG S2, PDF file, 0.4 MB. Copyright © 2020 Sharrar et al. 2020 Sharrar et al. This content is distributed under the terms of the Creative Commons Attribution 4.0 International license.

Novel clades basal to the extended class *Actinobacteria* were also recovered, two of which had members with significant numbers of KS and CD domains. These genomes are large (7 to 8 Mbp), with high GC content (>70%), and are divergent from existing publicly available actinobacterial genomes. The largest of these clades, likely a novel family in the *Streptosporangiales* order, contained the genome with the most BGCs (30 in total) found in the entire study (Streptosporangiales_ANG_2) (Fig. 2).

**FIG 4:**
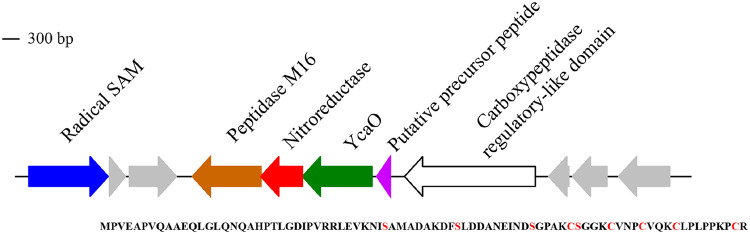
Diagram of a biosynthetic gene cluster predicted to produce a linear azole/azoline-containing peptide found in the candidate phyla radiation genome Microgenomates_ANG_785. The protein sequence for the putative precursor peptide is shown at the bottom with serine and cysteine residues likely modified by YcaO highlighted red.

Three BGCs were identified within CPR genomes. This was surprising, as, to our knowledge, clusters have rarely been reported in CPR genomes (24). The genome Microgenomates_ANG_786 encodes a lantipeptide, Saccharibacteria_ANG_806 encodes a terpene, and Microgenomates_ANG_785 encodes a biosynthetic gene cluster detected as a linear azole/azoline-containing peptide (LAP). LAPs belong to the family of ribosomally synthesized and posttranslationally modified peptide (RiPP) natural products and are defined by ribosomal synthesis of a precursor peptide and its subsequent posttranslational modifications (PTMs) (25). The gene cluster carries three PTM enzyme genes that are annotated as YcaO, nitroreductase, and peptidase (Fig. 4). A putative peptide precursor of 84 amino acids is present next to the YcaO gene. The YcaO product acts as a cyclodehydratase that modifies the Ser and Cys residues present in the core peptide region to azolines, which are subsequently oxidized to azoles by the flavin mononucleotide-dependent dehydrogenase encoded by the nitroreductase gene (26, 27). Finally, the peptidase is proposed to cleave the leader peptide region of the precursor peptide and release the natural product. We searched for similar LAP clusters in CPR by screening a data set enriched in CPR genomes (28). We found 110 LAP clusters in 93 additional CPR genomes (31 “*Candidatus* Microgenomates,” 51 *Parcubacteria*, 2 “*Candidatus* Peregrinibacteria,” and 1 “*Candidatus* Katanobacteria”), indicating that LAP clusters are more widespread than previously known in CPR. Most of them (89 LAP clusters) contained a nitroreductase gene next to the YcaO gene. While we can only predict the function of these clusters with the present data, a similar LAP cluster from a soil *Rhizobium* species was recently shown to target bacterial ribosomes with highly species-specific antibiotic activity (29).

### Biosynthetic potential with depth and vegetation.

**FIG 5:**
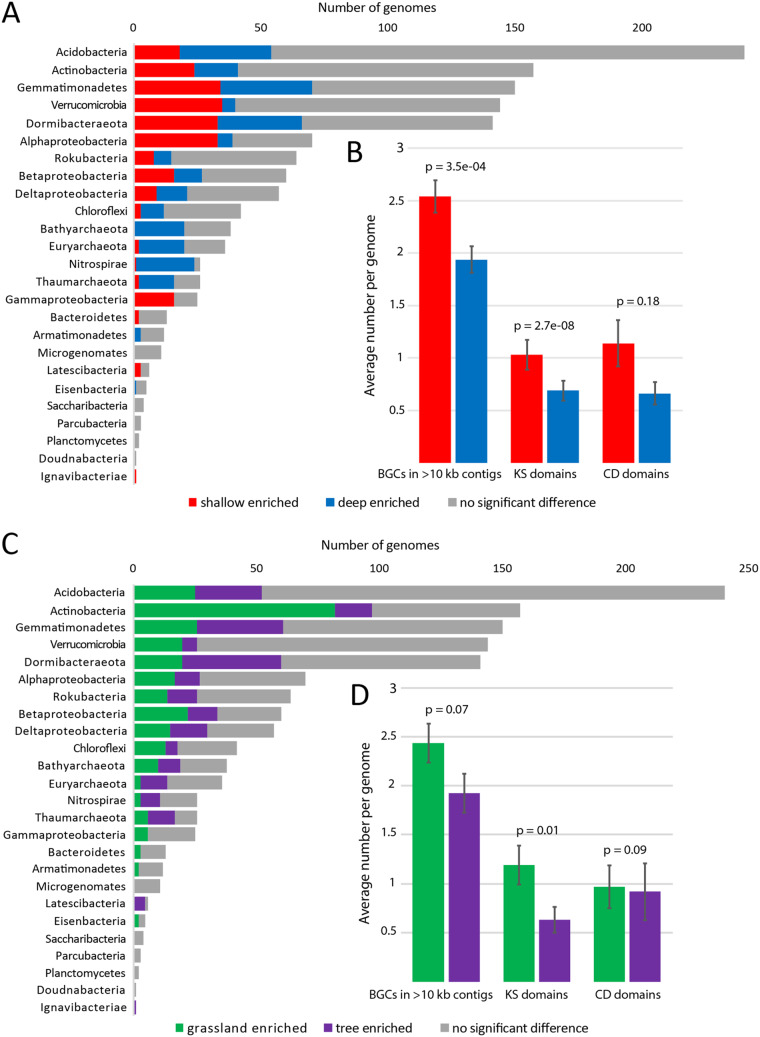
Genome, biosynthetic gene cluster (BGC), and key domain abundance with depth and vegetation. All *P* values are from Wilcoxon signed-rank tests, and error bars represent standard errors. (A) Number of genomes per taxonomic group that were deep enriched (blue), shallow enriched (red), or showed no significant change (gray) with depth, as determined by DESeq analysis of cross-mapped, dereplicated genomes (*n *= 1,334). (B) Average numbers of BGCs, KS domains, and CD domains per genome. (C) Number of genomes per taxonomic group that were enriched in grassland environments (green), enriched in tree-covered environments (purple), or showed no significant change (gray) with vegetation, determined like above. (D) Average numbers of BGCs, KS domains, and CD domains per genome.

Because depth was an important factor in microbial community composition (Fig. 1), biosynthetic potential with depth was investigated. Depth was treated as a continuous variable, and the trend of each organism’s abundance through the depths in which it was detected across samples was analyzed using DESeq2 (30). Among 1,334 organisms, 320 significantly increased in abundance with depth (were deep enriched) and 343 significantly decreased in abundance with depth (were shallow enriched) (false-discovery rate [FDR] of <0.05). Most taxonomic groups had members that were deep enriched or shallow enriched and that did not vary in abundance with depth (Fig. 5A). A few groups, such as members of *Archaea* and *Nitrospirae*, were primarily deep enriched, whereas *Gammaproteobacteria* were primarily shallow enriched.

Average numbers of BGCs, KS domains, and CD domains per genome were compared for genomes that were deep or shallow enriched. On average, genomes of organisms enriched in shallow samples encoded more BGCs and KS domains than genomes of organisms enriched in deep samples (Fig. 5B). The overall types of BGCs present in deep or shallow enriched genomes were not very different (Fig. S3).

10.1128/mBio.00416-20.3 FIG S3 Biosynthetic gene cluster (BGC) type abundance by soil environment. Percentages of BGC types within tree-enriched, grassland-enriched, deep-enriched, and shallow-enriched genomes are indicated. Known types with <1% overall abundance are grouped into the “Other” category. Download FIG S3, PDF file, 0.2 MB. Copyright © 2020 Sharrar et al. 2020 Sharrar et al. This content is distributed under the terms of the Creative Commons Attribution 4.0 International license.

In addition to depth, overlying vegetation type was tested for its significance in phylum selection and biosynthetic potential (Fig. 5C). According to DESeq2 analysis, 399 genomes were significantly enriched in grassland samples and 298 were significantly enriched in tree-covered samples. Overall, the types of BGCs present in genomes across vegetation classes were not very different (Fig. S3). However, on average, genomes that were enriched in grasslands encoded more KS domains than genomes that were enriched at tree-covered sites (Fig. 5D).

**FIG 6:**
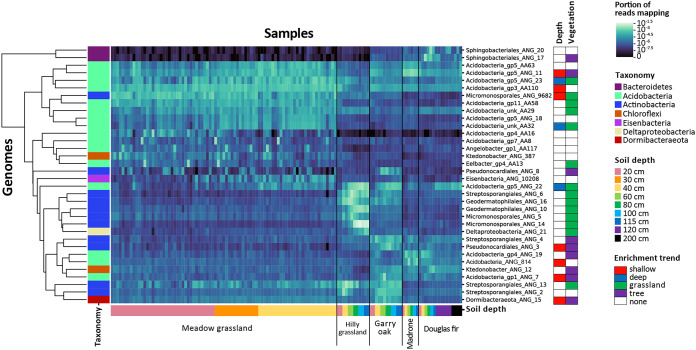
Abundance of genomes with highest biosynthetic potential across sampling sites and environments. All genomes from the dereplicated set (*n *= 1,334) with at least 15 total biosynthetic domains were included. Lighter heat map color, shown in log scale, indicates a higher portion of the reads in a sample (columns) mapping to a genome (rows). Genome rows were clustered based on similar abundance patterns. Genome taxonomy is shown in the left vertical colored bar. Samples are grouped by site and vegetation (black vertical lines) and then by soil depth (shown in horizontal colored bar). Environmental enrichment trends for each genome, as determined by DESeq, are shown in the “Depth” and “Vegetation” columns on the right.

In targeting members of particular phyla with high biosynthetic potential, it is important to consider how their abundance varies with environmental variables. Some of the genomes with at least 15 KS plus CD domains were found to be extremely abundant at certain sampling sites or soil depths and completely absent from others (Fig. 6). The genomes of *Acidobacteria* with the highest biosynthetic potential were generally prevalent in meadow grassland soil. *Actinobacteria* were more often enriched in grassland than in tree-covered soils, and those in the the subset with the highest biosynthetic potential were generally prevalent in hilly grassland soil. Within the *Chloroflexi*, Ktedonobacter_ANG_387 was more abundant in meadow grassland soil whereas Ktedonobacter_ANG_12 was more abundant at the other sites.

## DISCUSSION

### Understudied phylogenetic groups with high biosynthetic potential.

The *Actinobacteria* in this study were found to have some of the largest amounts of BGCs, KS, and CD domains in their genomes. *Actinobacteria* with high numbers of BGCs in this study were most often novel species within the class *Actinobacteria* (see Fig. S2 in the supplemental material; see also Table S4 in the supplemental material) and were often preferentially enriched in grassland relative to tree-covered (Garry oak) soil at the same site (Fig. 6). This extends findings of a prior 16S rRNA gene amplicon sequencing study by Charlop-Powers et al. (11) that correlated *Actinomycetales* abundance with high NRPS adenylation and KS domain richness in soil. Altogether, when targeting *Actinobacteria* and their biosynthetic products, vegetation type may be an important factor.

Another phylum exhibiting high biosynthetic potential was *Chloroflexi*. *Chloroflexi* are common in soil globally and are known for their large genomes, diverse morphologies, and complex lifestyles (31). They have been generally understudied regarding their biosynthetic potential; however, some in tropical forest soil (32) and marine sponges (33) were shown previously to encode a few PKS domains. In this study, Ktedonobacter_ANG_387 encoded 18 BGCs, with 14 classified as some type of NRPS or PKS or a hybrid combination. This level of enrichment is comparable to the highest degree of BGC enrichment previously shown in a few *Ktedonobacteria* genomes, with more NRPS/PKS clusters than had been reported previously (31). Screening of compounds produced by these *Ktedonobacteria* showed broad antimicrobial activity (31).

Understudied groups with notable biosynthetic potential include the candidate phyla “*Candidatus* Rokubacteria” and individual species of “*Candidatus* Eisenbacteria” and “*Candidatus* Dormibacteraeota.” “*Candidatus* Rokubacteria” was previously implicated in secondary metabolite production (16), but this function was not previously linked to “*Candidatus* Eisenbacteria” or “*Candidatus* Dormibacteraeota.” Although only five unique “*Candidatus* Eisenbacteria” genomes were recovered, one (Eisenbacteria_ANG_10208) encoded as many CD domains as some of the *Acidobacteria* and *Actinobacteria* genomes with the most CD domains. These results further emphasize that phyla not historically linked to secondary metabolite production may continue to prove to be sources of potentially pharmaceutically relevant compounds.

### Most common BGC types and their possible functions.

Although terpenes were the most abundant type of BGC overall, most of their ecological functions in bacteria remain poorly understood. It has been shown that bacteria can use some terpenes to communicate with each other and with fungi (34). Some terpenes also have antibacterial properties (35). Because many terpenes are volatile organic compounds, they have the advantage of being able to travel through both liquid-filled and air-filled soil pores, making them functional in a range of soil moistures. This trait may explain why they are so prevalent in these soils and in saprolites which experience large shifts in soil moisture throughout the year due to the Mediterranean climate and hydrogeologic effects (36). The wide range of novel terpene synthases in the diverse soil bacterial samples uncovered here remain to be characterized for their function and molecular products.

The next most abundant BGC type identified in this study was the combined group consisting of NRPS, PKS, and hybrid NRPS/PKS, which typically produce compounds such as antibiotics, antifungals, immunosuppressants, and iron-chelating molecules (8). After the NRPS/PKS clusters, the bacteriocins, which inhibit the growth of other microbes, were most prevalent overall. Bacteriocins are generally active against relatively closely related species and likely function in reducing competition in the same niche (35).

### Expanded phylogenetic ranges for some BGCs and possible functions.

An interesting observation was the presence of clusters implicated in production of ladderanes in *Verrucomicrobia*. While ladderane BGCs have previously been identified in *Streptomyces* (37), ladderanes are only known to be produced as components of the anammoxosome membranes of anammox bacteria (38). Anammox capabilities are known to be present only in *Planctomycetes* species, which are part of the PVC (*Planctomycetes*, *Verrucomicrobia*, and *Chlamydiae*) superphylum with *Verrucomicrobia* (39). The ladderanes uncovered here may serve unique, unknown functions.

We also recovered several novel BGCs for RiPPs (ribosomally synthesized and posttranslationally modified peptides) such as lassopeptides and lantipeptides. Lassopeptide BGCs were newly found in “*Candidatus* Latescibacteria” and *Armatimonadetes* genomes. Lassopeptides can have antimicrobial, enzyme-inhibitory, and receptor-antagonistic activities (40). Further, lantipeptides are known to be widespread phylogenetically (41), but this is the first time that a cluster has been reported in a CPR bacterial genome. As lantipeptides can include lantibiotics, the finding is notable given that metabolic reconstructions performed for CPR bacteria have consistently predicted them to be symbionts (42).

Indoles have many functions, including disruption of quorum sensing and virulence capabilities of plant pathogens and control of plant growth and root development (35). In this study, high proportions of indole BGCs were found in *Gammaproteobacteria* and in some genomes of bacteria from the newly named candidate phylum “*Candidatus* Dormibacteraeota.” Interestingly, one indole BGC was also found encoded in a “*Candidatus* Bathyarchaeota” genome.

Phosphonates are known to be widespread among microbes, as some have been found in *Archaea* (43). While none of the few archaeal BGCs identified in this study were classified as phosphonates, phosphonate BGCs were particularly abundant in *Nitrospirae*, which, like *Archaea*, typically increase in relative abundance with soil depth. Phosphonates are known to function as antibacterials, antivirals, and herbicides. They also provide a mechanism to store phosphorus, which can sometimes be scarce and limiting (43). Phosphonate use may be an adaptation of the *Nitrospirae* for survival in deep soil and saprolite.

### Biosynthetic capacity varies with depth and vegetation.

Our finding that bacteria in shallow soil had on average higher biosynthetic capabilities than bacteria in deep soil may be attributed to the greater opportunities for interaction and competition in shallow soils, where microbial biomass and diversity are higher (44, 45). We also found that biosynthetic potential varied with vegetation type within a local environment. Some secondary metabolites, such as plant growth hormones and certain antibiotics, are produced by bacteria to benefit specific plants in their environment (46). Previously, it was demonstrated that the biosynthetic potential of amplified KS domains varies with vegetation on the continental scale (13), and here we demonstrate similar patterns on a local scale without PCR biases.

Abundances of the different types of BGCs were relatively consistent across genomes differentially enriched by either depth or vegetation. Few studies have been published comparing levels of biosynthetic potential across environments. However, one recent study similarly found that bacteriocin, NRPS/PKS, and terpene clusters were the most common BGC types in 30 genomes of soil bacteria from different environments (35). These findings suggest that while the distribution of broad types of BGCs is mostly consistent across soil environments, the amounts of PKS and NRPS gene clusters may be dependent on environment.

### Conclusion.

Genome-resolved metagenomics of environmental samples allows the discovery of new biosynthetic gene clusters and determination of the organisms and ecosystems that they reside in. Here, we uncovered environmental controls of the distribution of biosynthetic gene clusters associated with bacteria that vary in abundance with soil depth and vegetation type. This information will be useful for researchers of natural products who wish to clone, isolate, or sequence the genes of these clusters. Notably, we have broadened the range of phylogenetic targets for microbial products of interest, especially of NRPs and PKs. Microbial products have obvious utility in medicine and biotechnology, but they are also important for their effects on microbial communities and biogeochemical cycles. There remains much to discover about the nature of diverse secondary metabolisms in the environment.

## MATERIALS AND METHODS

### Sampling sites.

Soil and saprolite samples were taken in areas studied by the Eel River Critical Zone Observatory (CZO). Samples were taken from soil depths of 20 to 200 cm over a 4-year period, from 2013 to 2016. The Eel River CZO experiences a Mediterranean climate characterized by hot, dry summers and cool, wet winters. The first fall rain after the dry summer generally comes in middle to late September, and most rain falls between November and March. Average yearly rainfalls range from about 1.8 to 2 cm (47).

**FIG 7:**
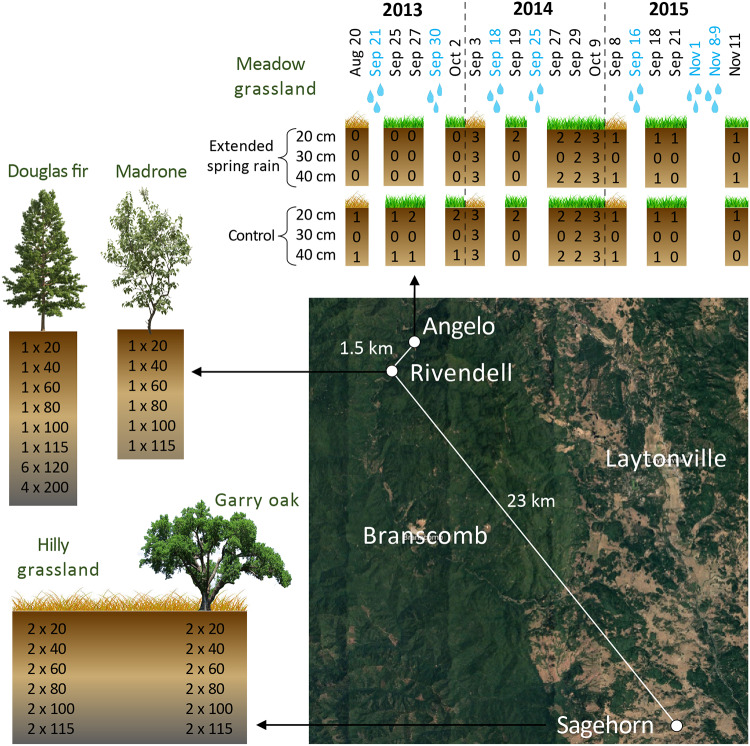
Eel River CZO sampling scheme. Soil and saprolite samples were taken from depths of 20 to 200 cm across three sites between 2013 and 2016. At Angelo, meadow grassland samples were taken before and after the first fall rains in 2013 to 2015 on the dates shown (blue = natural rainfall events). Numbers in the boxes show how many samples were taken at each depth on each date from either control plots or plots with experimentally extended spring rainfall. At Rivendell, samples were taken from both the north slope, under a Douglas fir tree, and the south slope, under a Madrone tree. Numbers in the boxes show how many samples were taken × depth (cm). Similarly, at Sagehorn, samples were taken from below a Garry oak tree and in the nearby hilly grassland. Aug, August; Sep, September; Oct, October; Nov, November. (Map data © 2018 Google.)

Samples were collected at two sites within the Angelo Coast Range Reserve: Rivendell, which is a forested hillslope (48, 49), and a nearby meadow (17, 18, 50). The meadow and Rivendell are 1.5 km apart (Fig. 7). Both are underlain by the Coastal Belt of the Franciscan Formation, which consists of mostly argillite (shale), with some sandstone and conglomerate (51). At Rivendell, the soil mostly lacks distinct horizons (36) and varies in depth from 30 to 75 cm, with saprolite directly below (47). The northern slope of Rivendell is dominated by Douglas fir (Pseudotsuga menziesii) trees, while the southern slope has more Pacific madrone (Arbutus menziesii) trees. In the Angelo meadow, grass roots are confined to depths of <10 cm (17).

A third study site, Sagehorn, is a hilly grassland located about 23 km to the southeast of the other two sites (Fig. 7) (52, 53). Sagehorn is underlain by the Central Belt of the Franciscan Formation, a mélange with a sheared argillaceous matrix containing blocks of sandstone and other lithologies (54). Sagehorn soils generally have a 30-cm-thick organic-rich horizon underlain by a 10-to-20-cm-thick clay-rich horizon, directly above saprolite (53). The low-porosity mélange bedrock causes these layers to become entirely saturated in the winter wet season (53). Sagehorn is primarily a grassland with scattered Garry oak (Quercus garryana) trees.

### Sampling and DNA extraction.

At the meadow sites, 10 samples were taken on four dates in 2013 spanning periods before and after the first two fall rain events at a soil depth of either 20 or 40 cm, as described in a previous publication (17). In 2014, 60 samples were taken on five dates before and after the first two fall rain events at soil depths of 20, 30, and 40 cm. Samples came from six different plots, including three treatment plot replicates with artificially extended spring rain and three control plot replicates, as described by Diamond et al. (18). The extended spring rain plots received supplemental water from April to June (when there is very little natural rain) each year from 2001 through 2015 (50). In 2015, 13 samples were taken on four dates spanning the periods before and after the first few fall rain events at a soil depth of either 20 or 40 cm on either a control or treatment plot (Fig. 7). All Angelo samples are referred to here as “meadow grassland” samples.

At Rivendell, a depth profile of six samples (from depths of 20, 40, 60, 80, 100, and 115 cm) was taken on the Douglas fir-dominated northern slope in 2013. Sterile scoops were used to sample soil and saprolite from a bucket auger. Samples were scooped directly into sterile Whirl-Pak bags and flash frozen on site in dry ice and ethanol. In 2015, 10 deep saprolite samples were taken from the northern slope. A trackhoe outfitted with a coring auger was used to drill into the hillslope saprolite beneath mature Douglas fir trees. At depths of 120 and 200 cm, samples were taken using a sterilized hand auger. All samples from the northern slope of Rivendell are referred to here as the “Douglas fir” samples. In 2016, a similar depth profile of six samples (collected from depths of 20 to 115 cm) was taken on the southern slope under a Pacific madrone tree (the “Madrone” samples). A soil pit was dug using a jackhammer, the wall of the pit was sampled with sterile scoops, and the samples were placed into 50-ml Falcon tubes which were immediately flash frozen on dry ice.

At Sagehorn, a depth profile of 12 samples (2 samples each at depths of 20, 40, 60, 80, 100, and 115 cm) was taken from under a Garry oak tree (the “Garry oak” samples) and from the grassland (the “hilly grassland” samples) approximately 10 m away, for a total of 24 samples. The two soil pits were dug using a jackhammer. The walls of the pits were sampled on both sides with a sterile scoop, resulting in two samples per soil depth collected approximately 10 cm apart laterally. Samples were scooped into sterile 50-ml Falcon tubes which were immediately flash frozen on dry ice.

All samples were transported on dry ice and stored at –80°C until DNA extraction. In all cases, DNA was extracted from 10 g of material with a MoBio Laboratories PowerMax soil DNA isolation kit, using a previously described protocol (17). This resulted in a total of 129 metagenomic samples across the Eel River CZO (see Table S1 in the supplemental material).

10.1128/mBio.00416-20.4 TABLE S1 Metadata for 129 metagenomic soil and saprolite samples. Download Table S1, XLSX file, 0.1 MB. Copyright © 2020 Sharrar et al. 2020 Sharrar et al. This content is distributed under the terms of the Creative Commons Attribution 4.0 International license.

10.1128/mBio.00416-20.5 TABLE S2 Genome information, antiSMASH data, and DESeq2 results for the 1,334 genomes used in this study. Download Table S2, XLSX file, 0.2 MB. Copyright © 2020 Sharrar et al. 2020 Sharrar et al. This content is distributed under the terms of the Creative Commons Attribution 4.0 International license.

10.1128/mBio.00416-20.6 TABLE S3 Biosynthetic gene clusters included in this study. Download Table S3, XLSX file, 0.1 MB. Copyright © 2020 Sharrar et al. 2020 Sharrar et al. This content is distributed under the terms of the Creative Commons Attribution 4.0 International license.

10.1128/mBio.00416-20.7 TABLE S4 Genome information and closest 16S or ribosomal protein L6 hits of *Actinobacteria* genomes included in Fig. S2. Download Table S4, XLSX file, 0.04 MB. Copyright © 2020 Sharrar et al. 2020 Sharrar et al. This content is distributed under the terms of the Creative Commons Attribution 4.0 International license.

### DNA sequencing and assembly and genome reconstruction.

All metagenomic library preparation and DNA sequencing procedures were done at the Joint Genome Institute. Douglas fir samples collected in 2013 and meadow grassland samples collected in 2013 to 2014 were sequenced using 250-bp paired-end Illumina reads. Meadow grassland reads from samples collected in 2014 were quality trimmed to 200 bp and assembled into individual metagenomes using a combination of IDBA-UD (55) and MEGAHIT (54), as previously described (18). All other metagenomes were sequenced using 150-bp paired-end Illumina reads and data sets individually assembled using IDBA-UD (55). Open reading frames were predicted with Prodigal (56) and annotated by using USEARCH (57) to search for similarity against the UniProt (58), UniRef90, and KEGG (59) databases.

This data set included genomes binned from prior studies (17, 18) and newly reported genomes. Newly reported genomes from 2015 meadow grassland samples were binned using differential coverage binners ABAWACA2 (60), MaxBin2 (61), CONCOCT (62), and MetaBAT (63). Scaffolds from all other metagenomes were binned using ABAWACA2 (60), MaxBin2 (61), and MetaBAT (63). For all metagenomes binned with multiple automated binners, the highest-quality bins from each metagenome were selected using DasTool (64).

### Ribosomal protein S3 (rpS3), ordination, and variable importance analysis.

All proteins predicted from the 129 metagenomes were searched for ribosomal protein S3 (rpS3) sequences using a custom hidden Markov model (HMM) from Diamond et al. (18) with a score threshold of 40. Only rpS3 proteins with lengths in the 120-to-450-amino-acid range were considered, resulting in 20,789 rpS3 proteins. RpS3 protein taxonomy was identified at the phylum level using USEARCH (57) to search against a database of rpS3 proteins from Hug et al. (65) with an E value threshold of 1e−10. RpS3 proteins were then clustered at 99% amino acid identity using USEARCH. This resulted in 7,013 dereplicated rpS3 sequences, each representing an approximately species-level cluster. Reads from each sample were mapped against the largest rpS3-containing scaffold in each cluster using Bowtie2 (66). Read mappings were filtered for ≥98% sequence identity, and a coverage table was created by calculating coverage per base pair. The coverage table was normalized for sample sequencing depth using the following formula: (coverage/reads input to the sample’s assembly) × average number of reads input to assemblies. The coverage table was used as input to the nonmetric multidimensional scaling (NMDS) ordination and variable importance analysis performed in R using the vegan package (67). First, Bray-Curtis dissimilarities were calculated using the vegdist command. Then the NMDS was performed using these dissimilarities with the metaMDS command and the following options: k = 3, try = 500, trymax = 500 (NMDS stress = 0.0597). The relative importance of metadata variables for community composition was calculated through permutational multivariate analysis of variance (PERMANOVA) using the adonis2 command with the following formula and options: formula = ∼site + depth + vegetation, by = “margin,” permutations = 9999.

### Genome filtering and dereplication.

Bins were initially filtered for completeness and contamination based on the inventory of 38 archaeal single-copy genes or 51 bacterial single-copy genes, except for CPR bacteria, where a reduced set of 43 CPR-specific genes was used (68). Bins that had at least 70% of the single-copy genes in their respective sets with <4 having multiple copies were kept in the analysis. Next, CheckM (69) lineage_wf was run on these bins, with a threshold of >70% complete with <10% contamination (for non-CPR bins only). To achieve the final draft genome set, bins were dereplicated at 98% nucleotide identity using dRep (70).

### Tree building and taxonomic determination.

Genomes with >50% of their genes annotated to have best hits in one phylum were automatically assigned to that phylum. To check this phylum classification and identify the remaining genomes, a maximum-likelihood tree was calculated based on the concatenation of 16 ribosomal proteins (L2, L3, L4, L5, L6, L14, L15, L16, L18, L22, L24, S3, S8, S10, S17, and S19). Sequences were aligned using MAFFT (71) version 7.390 (–auto option). Each alignment was further trimmed using trimAl (72) version 1.4.22 (–gappyout option) before being concatenated. Tree reconstruction was performed using IQ-TREE (73) version 1.6.6 (as implemented on the CIPRES Web server [74]) and ModelFinder (75) to select the best model of evolution (LG+F+I+G4), and with 1,000 ultrafast bootstrap replicates. Using the same method, but with model LG+I+G4, a tree was created to compare *Actinobacteria* in this study to NCBI references from each *Actinobacteria* genus. All trees were visualized with iTol (76).

### Differential abundance analysis.

Reads from all 129 metagenomes were mapped against the dereplicated set of genomes using Bowtie2 (66). Raw read counts for each genome across each sample were input into DESeq2 (30) using R. Differential abundance across depth, controlling for site, was tested using the DESeq2 model as follows: design = ∼site + depth. Each genome with a *P* value adjusted for a false-discovery rate (FDR) of <0.05 for either increasing with depth (deep enriched) or decreasing with depth (shallow enriched) was put into its respective category. Differential abundance with vegetation was tested using the DESeq2 model as follows: design = ∼site + vegetation. Vegetation was classified simply as either grassland (meadow and hilly grassland samples) or tree covered (Douglas fir, Madrone, and Garry oak samples). Each genome with an FDR adjusted *P* value of <0.05 for either tree covered or grassland enriched was put into the respective category.

### Biosynthetic gene cluster (BGC) analysis.

To identify biosynthetic gene clusters (BGCs), antiSMASH 4.0 (77) was run on the final dereplicated set of genomes using default parameters. Only BGCs on contigs of >10 kb were considered. Ketosynthase (KS) and condensation (CD) domains were identified using Pfam (78) HMMs PF00109 and PF00668, respectively. BGC type was determined from the antiSMASH output. Only types that made up at least 1% of all BGCs are named in the figures; the remainder were classified in the “Other” category. This category is made up both of types present at <1% and of BGCs that antiSMASH could not confidently place into a type category. The Wilcoxon signed-rank test was used in R to calculate significant differences between the average numbers of BGCs, KS domains, and CD domains per genome for groups of genomes showing enrichment with depth or vegetation.

### Data availability.

Sequencing reads and assembled sequences are available for the 2013 and 2014 meadow grassland samples under NCBI BioProject accession numbers PRJNA297196 and PRJNA449266, respectively. Sequencing reads and assembled sequences for all other samples are available under NCBI BioProject accession number PRJNA577476. All genome sequences are available at https://doi.org/10.6084/m9.figshare.10045988.
