# Supplementary figures and images for: Bacterial Secondary Metabolite Biosynthetic Potential in Soil Varies with Phylum, Depth, and Vegetation Type
Source: mBio. 2020 Jun 16;11(3):e00416-20. doi: 10.1128/mBio.00416-20 (PMC7298704; doi:10.1128/mBio.00416-20)

Tree scale: 0.1

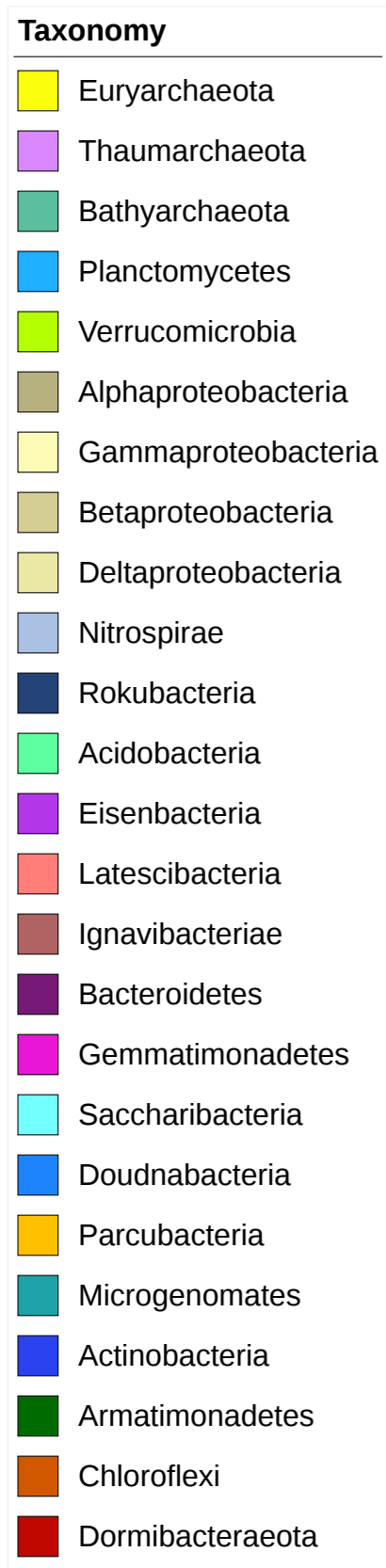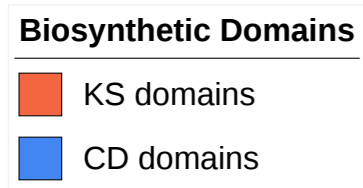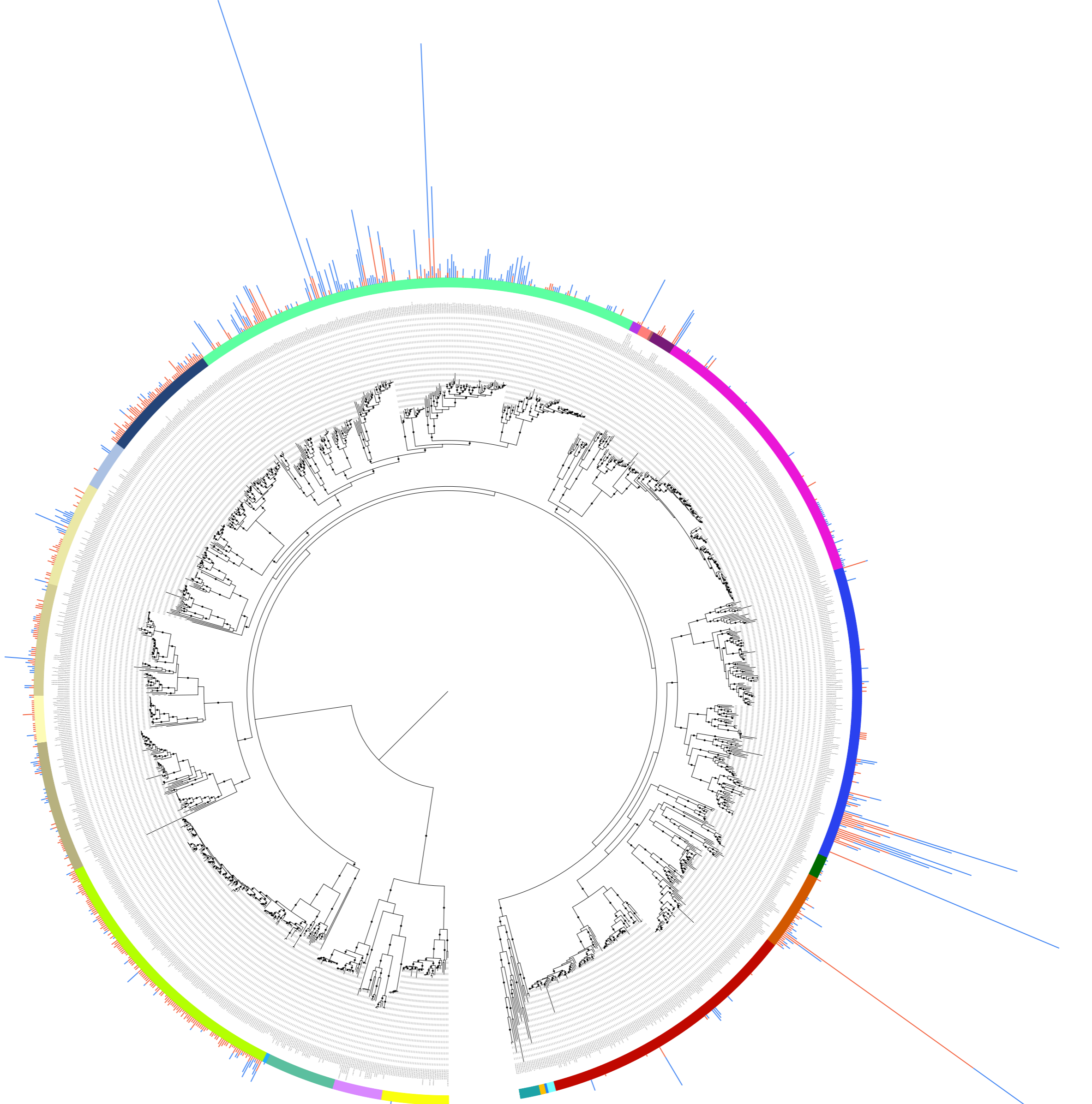

Supplement: FIG S1 [file mBio.00416-20-sf001.pdf]

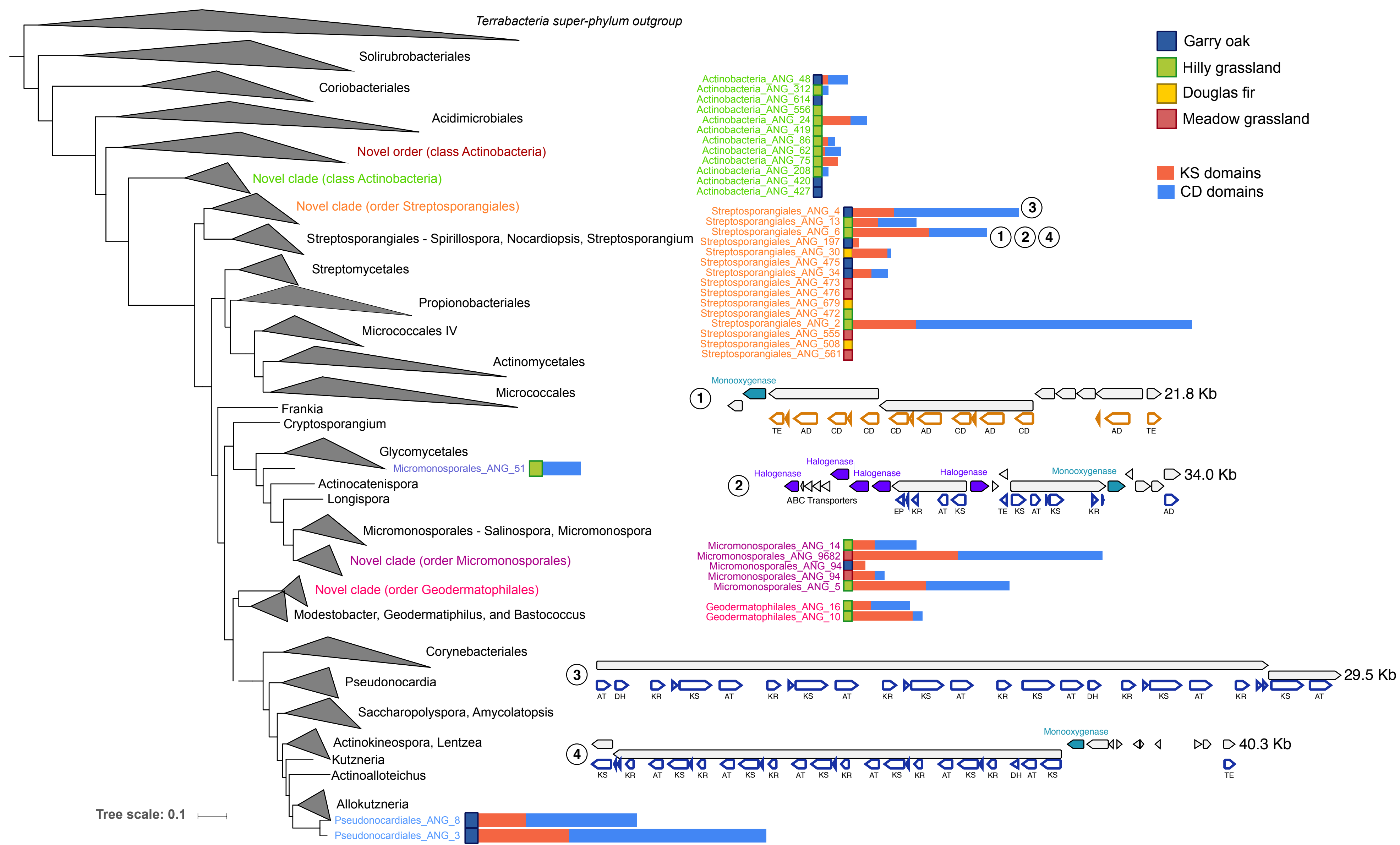

Supplement: FIG S2 [file mBio.00416-20-sf002.pdf]

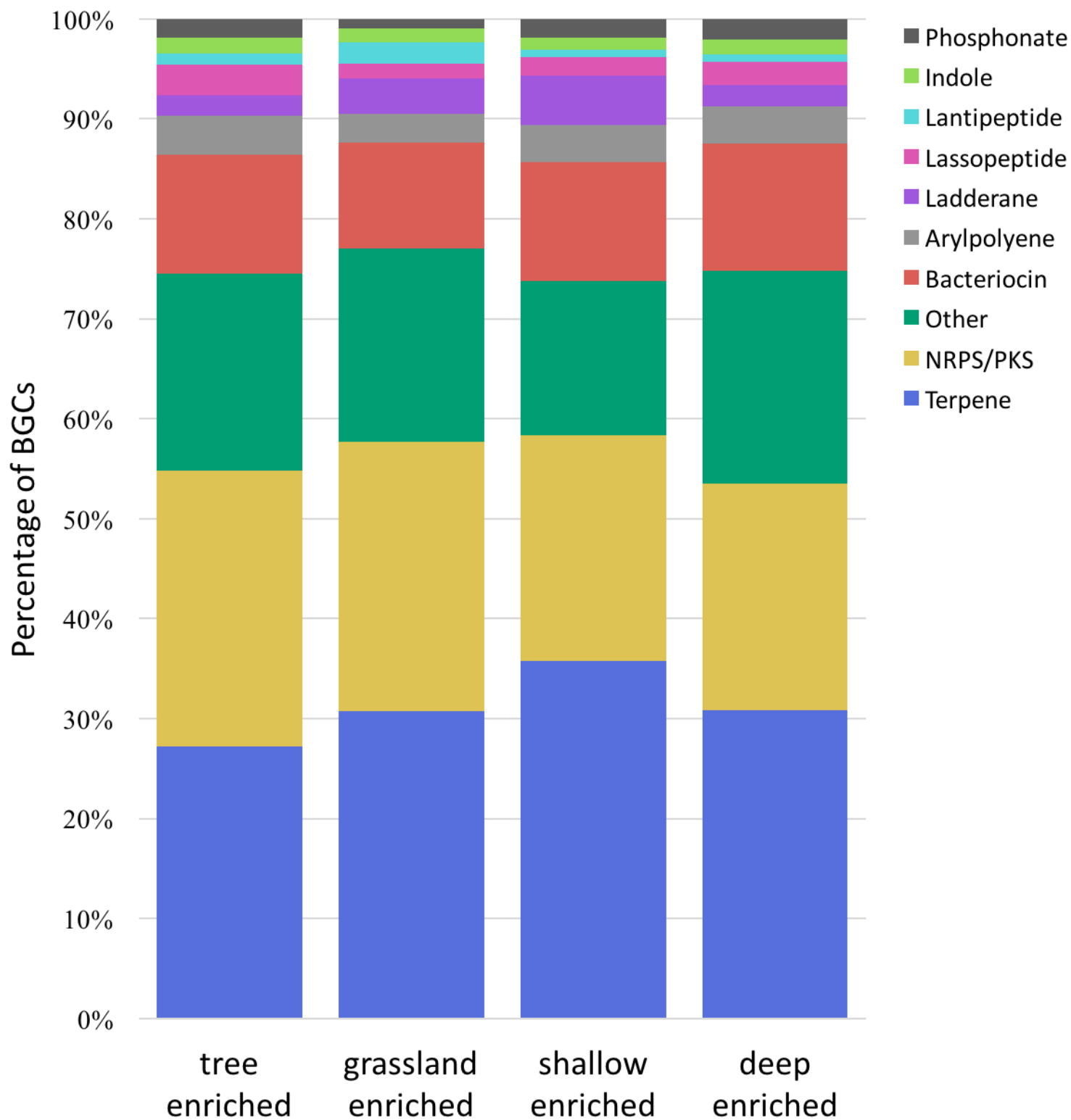

Supplement: FIG S3 [file mBio.00416-20-sf003.pdf]
